# Timing matters: gastrostomy placement and outcomes in patients with head and neck cancer undergoing chemoradiotherapy

**DOI:** 10.3389/fnut.2026.1885057

**Published:** 2026-09-18

**Authors:** Esther Forkosh, Yousra Sharif, David Hakimian

**Affiliations:** 1 Hadassah Medical Center, Institute of Gastroenterology and Hepatology, Jerusalem, Israel; 2 Institute of Gastroenterology and Hepatology, Hadassah Medical Center, Jerusalem, Israel

**Keywords:** chemoradiotherapy, head and neck cancer, nutritional support, prophylactic gastrostomy, survival outcomes

## Abstract

**Background:**

Patients with head and neck cancer (HNC) undergoing chemoradiotherapy (CRT) are at high risk of malnutrition. The optimal timing of gastrostomy [percutaneous endoscopic gastrostomy (PEG) or radiologically inserted gastrostomy (RIG)] placement remains controversial.

**Objective:**

To compare survival, nutritional outcomes, and gastrostomy-related complications between prophylactic and later gastrostomy placement.

**Methods:**

In this retrospective TriNetX US Collaborative Network cohort, prophylactic gastrostomy (within ±14 days of CRT initiation) was compared with early-reactive (15–90 days), late-reactive (91–180 days), and very-late (>180 days) placement using 1:1 propensity-score matching on demographic, clinical, and laboratory characteristics. Survival was measured from index gastrostomy; therefore, comparisons with later placement were conditional on survival until gastrostomy and considered exploratory rather than estimates of the effect of placement timing.

**Results:**

The prophylactic-versus-very-late comparison included 2,251 matched pairs, analyzed at 1–2 and 2–5 years. Post-gastrostomy survival was similar between prophylactic and early-reactive placement at both intervals [hazard ratio (HR) 0.94 and 0.95; both nonsignificant]. Compared with very-late placement, survival did not differ at 1–2 years (HR 0.90, 95% CI 0.65–1.25) but was higher at 2–5 years (88.4% vs. 83.2%; HR 0.69, 95% CI 0.48–0.98); adjusted HRs were 0.72 (0.54–0.96) and 0.61 (0.45–0.83), respectively. Survival was higher with prophylactic versus late-reactive placement at 1–2 years (84.2% vs. 77.8%; HR 0.70, 95% CI 0.54–0.90), but not at 2–5 years (HR 0.64, 95% CI 0.32–1.26). Prophylactic placement was associated with fewer gastrostomy-related complications than very-late placement (1.8% vs. 3.1% at 1–2 years; 1.3% vs. 2.6% at 2–5 years), less aspiration pneumonia at 1–2 years (1.0% vs. 1.9%), and higher serum albumin (3.89 vs. 3.70 g/dL; all *p* < 0.05), while malnutrition (*p* = 0.10) and body mass index (*p* = 0.45) did not differ.

**Conclusion:**

Prophylactic and early-reactive placement had comparable post-gastrostomy survival, supporting early-reactive placement as a reasonable alternative in appropriately monitored patients. Poorer outcomes with late and very-late placement may identify a clinically higher-risk population rather than demonstrate a benefit of earlier placement. Prospective target-trial emulation studies are needed to define optimal timing.

## Introduction

Head and neck cancer (HNC), encompassing malignancies of the oropharynx, hypopharynx, larynx, and oral cavity, is the seventh most common cancer worldwide (1) and is frequently managed with definitive chemoradiotherapy (CRT), particularly in locally advanced, unresectable disease (2). CRT is frequently complicated by treatment-related toxicities, including mucositis, dysphagia, and odynophagia, which impair oral intake (3, 4). Malnutrition is therefore among the most frequent complications, with its prevalence rising from roughly 10% at baseline to up to 74% by the end of treatment (5–7). During CRT, malnutrition reduces tolerance to therapy, forces unplanned treatment interruptions (8), and worsens toxicity (6, 9); failure to regain baseline weight independently predicts poor overall survival (8, 10, 11).

To mitigate these risks, a gastrostomy (either percutaneous endoscopic gastrostomy [PEG] or radiologically inserted gastrostomy [RIG]) is often placed before or early during CRT to provide enteral nutritional support (12). Gastrostomy placement is generally safe. Reported risks include infection, tube dislodgement, leakage, and long-term tube dependency, although severe complications are uncommon and procedure-related mortality is below 1% (13–16). Its use therefore requires balancing procedure-related morbidity against the consequences of untreated malnutrition.

Nutritional guidelines from the European Society for Clinical Nutrition and Metabolism (ESPEN) and the American Society for Parenteral and Enteral Nutrition (ASPEN) recommend initiating enteral feeding when oral intake or nutritional status is inadequate (17, 18). However, neither specifies the optimal timing of prophylactic gastrostomy placement (19). Studies addressing this question are limited by small sample sizes, single-center designs, and selection bias (20).

To address this knowledge gap, we used the TriNetX federated electronic health record network to perform a large PSM cohort study comparing overall survival, nutritional outcomes, and gastrostomy-related complications across prophylactic, early-reactive (15–90 days), late-reactive (91–180 days), and very-late (>180 days) gastrostomy placement following CRT initiation.

## Materials and methods

We conducted a retrospective, multicenter cohort study on the TriNetX US Collaborative Network (Cambridge, MA), a federated network providing de-identified electronic health record (EHR) data from over 100 million patients across 66 US healthcare organizations, predominantly academic medical centers and community hospitals (21–24). The analysis was performed on June 27, 2026; the network is distributed, with analytics run at each organization and only aggregate, de-identified results returned, in compliance with the Health Insurance Portability and Accountability Act (HIPAA) (TriNetX is ISO 27001:2013 certified). Access was obtained through an institutional subscription held by Hadassah Medical Center under a data-use agreement; the data are not publicly downloadable, but all diagnostic, procedural, and laboratory codes and the full cohort-definition and matching criteria are reported herein, enabling reproduction by any investigator with TriNetX access. The study is reported following the Reporting of studies Conducted using Observational Routinely-collected health Data (RECORD) statement (25). The completed checklist is provided in Supplementary material.

### Ethics

The TriNetX US Collaborative Network data are de-identified by HIPAA Privacy Rule §164.514(b)(1) expert determination and analyzed only as aggregated summaries containing no protected health information. The study therefore did not constitute human subjects research, was exempt from institutional review board approval, and did not require informed consent. It was conducted in accordance with the Declaration of Helsinki.

### Study population

Adult patients (≥18 years) with HNC (ICD-10 C00–C14, C32) who received concurrent CRT between January 1, 2010, and June 27, 2026 were identified. Patients with a pre-existing gastrostomy placed at least 1 month before CRT initiation, or with undeterminable gastrostomy timing, were excluded. Eligible patients were stratified by the timing of their index gastrostomy relative to CRT into four groups: prophylactic (within ±14 days), early-reactive (15–90 days), late-reactive (91–180 days), and very-late (>180 days). As no standardized timing thresholds exist in HNC (19), these intervals were investigator-defined as descriptive timing categories relative to CRT initiation. Classification was based solely on the elapsed time between CRT initiation and gastrostomy placement; the database did not reliably capture the clinical indication, urgency, or intended feeding strategy. Consequently, late and very-late placement should not be interpreted as delayed versions of the same planned intervention. These placements may have represented rescue nutritional access in patients with disease recurrence or progression, treatment-related fibrosis or stenosis, progressive dysphagia, functional decline, or palliative-care needs. Prophylactic placement was compared separately with the early-reactive, late-reactive, and very-late cohorts.

### Covariates and matching

Baseline covariates comprised demographic [age, sex, and self-reported race (e.g., White, Black, Asian) and ethnicity (Hispanic vs. non-Hispanic), recorded as separate fields in TriNetX and both retained to optimize matching balance]; comorbidities (hypertensive disease, ischemic heart disease, heart failure, atrial fibrillation/flutter, cerebrovascular disease, malignant neoplasms of the lip/oral cavity/pharynx, malignant neoplasms of ill-defined/secondary/unspecified sites, tracheostomy status, and malnutrition); primary tumor subsite and cancer stage; and laboratory and functional parameters [serum albumin, C-reactive protein, and Eastern Cooperative Oncology Group (ECOG) performance status]. ECOG performance status was prespecified as a matching covariate; however, because it was recorded for only 0.5% of patients, it contributed limited information to propensity-score estimation. Comorbidities were selected *a priori* as prevalent conditions with prognostic relevance in HNC and for CRT tolerance (26, 27). The complete diagnostic, procedural, and laboratory codes are provided in Supplementary Table S1. Formal cancer stage was documented in only a minority of patients. The heterogeneous C76–C80 code block, which includes ill-defined, secondary, and unspecified malignant sites, was retained only as a recorded diagnostic covariate. It was not considered a surrogate for anatomical TNM stage or tumor burden and could not distinguish regional involvement from distant metastatic disease. Consequently, cancer stage and disease burden remained inadequately adjusted in the matched comparisons. Cohort entry required a primary HNC code (C00–C14 or C32); therefore, C76–C80 codes were not used to define the study population. To minimize confounding, 1:1 PSM was performed using a greedy nearest-neighbor algorithm with a caliper of 0.1 pooled standard deviations. Covariate balance was assessed using standardized mean differences (SMDs), with values <0.10 indicating adequate balance.

### Outcomes

The primary outcome was overall survival, assessed by Kaplan–Meier estimation and Cox proportional hazards regression. Secondary outcomes were diagnosis of malnutrition, nutritional parameters (body mass index [BMI] and serum albumin), gastrostomy-related complications, aspiration pneumonia, and emergency department visits; the corresponding diagnostic, procedure, and laboratory codes are listed in Supplementary Table S1. Outcomes were evaluated within two prespecified follow-up intervals after the index gastrostomy: 1–2 years (365–730 days) and 2–5 years (730–1825 days). Matching was performed separately for each timing comparison and each follow-up interval; because the federated network is queried at run time, cohort sizes varied slightly between analyses.

### Statistical analysis

Analyses were performed on the TriNetX Analytics platform, which uses validated Java, R, and Python software; further details of the platform and its statistical methods have been published previously (28). Continuous variables (among patients with available measurements) were compared with t-tests (robust to non-normality at these sample sizes) and categorical variables with chi-square tests, reporting mean differences and risk ratios with 95% CIs. Survival was assessed by Kaplan–Meier estimation with log-rank tests and Cox proportional-hazards models [hazard ratios (HRs) with 95% CIs; proportionality verified]. Imbalance in measured covariates was addressed using two complementary approaches: propensity-score matching for the Kaplan–Meier and risk-based analyses, and multivariable Cox regression adjusted for age, sex, and primary tumor site. These methods did not address survivor-selection related to gastrostomy timing or residual confounding from unmeasured or incompletely recorded characteristics. As the study included all eligible patients, the sample size was fixed and no prior power calculation was required; significance was defined by the conventional two-sided threshold of *p* < 0.05, and secondary outcomes were prespecified without adjustment for multiple comparisons. For all survival analyses, time zero was the date of the index gastrostomy (the point of exposure). PSM adjusted only for recorded covariates and did not correct the survivor-selection created by requiring patients to survive until gastrostomy placement. Consequently, this bias was not quantitatively eliminated, and the survival analyses were interpreted as conditional, exploratory associations. Patients with outcomes before a follow-up window were retained rather than excluded, with censoring at the last recorded activity. A patient could enter a later group only by surviving long enough to receive the gastrostomy at that timing, so each group consists of survivors to that point. These analyses therefore estimate survival from gastrostomy placement within each timing group rather than the effect of a feeding strategy assigned at the start of CRT.

## Results

Of 287,807 patients with HNC, 117,491 underwent CRT and 27,832 received a gastrostomy; after excluding those with a pre-existing gastrostomy or undeterminable timing (*n* = 16,676), the remaining patients were classified as prophylactic (*n* = 4,345), early-reactive (*n* = 3,110), late-reactive (*n* = 1,187), or very-late (*n* = 2,514). After 1:1 PSM, the matched prophylactic–very-late comparison comprised 2,251 pairs, analyzed during the 1–2- and 2–5-year intervals after gastrostomy placement (Figure 1).

**Figure 1:**
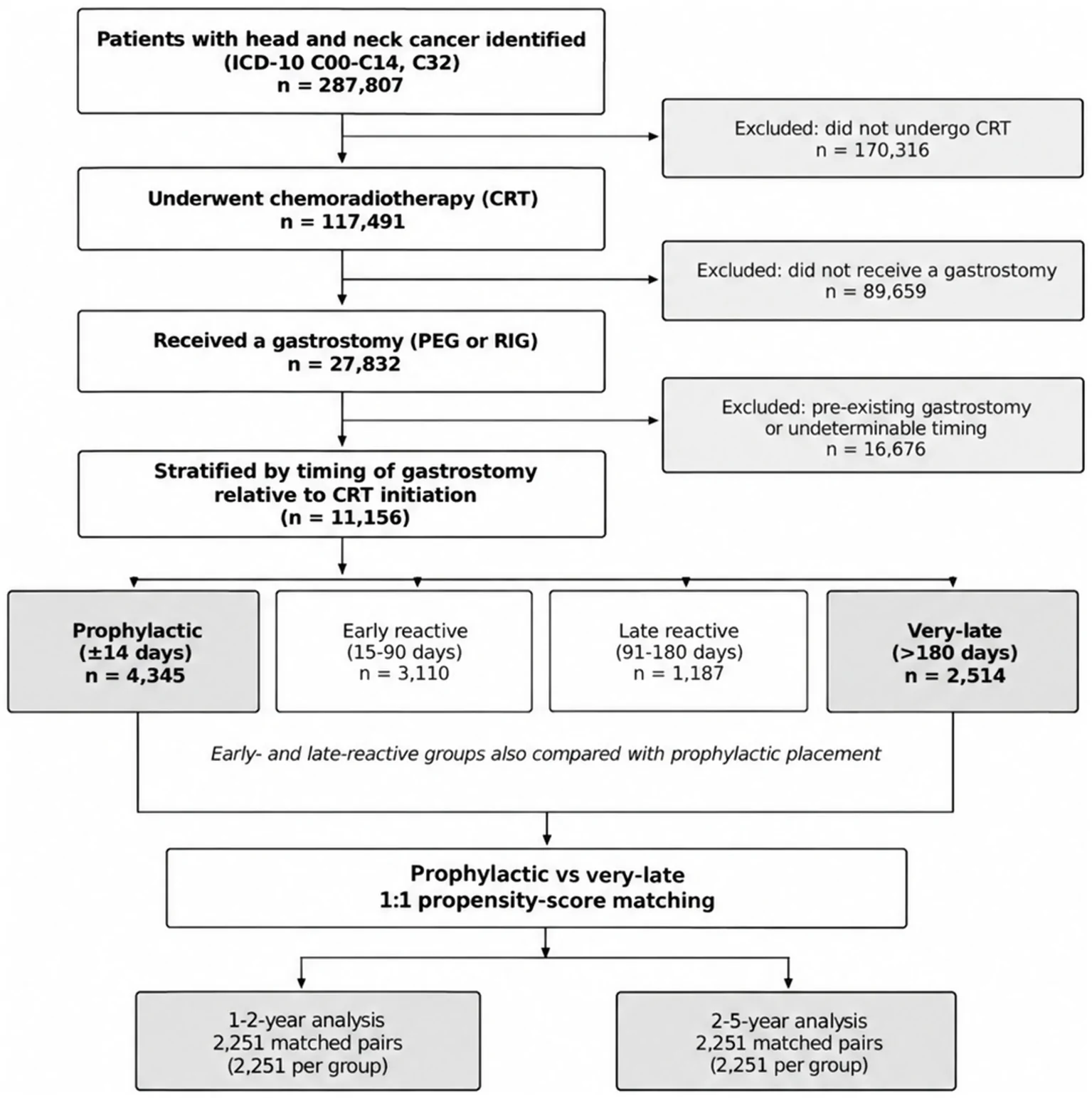
Study flow and stratification of patients with head and neck cancer by timing of gastrostomy relative to chemoradiotherapy. Shaded boxes denote the matched prophylactic and very-late cohorts, which underwent 1:1 propensity-score matching (2,251 patients per group). Prophylactic placement was additionally compared with the early- and late-reactive groups. CRT, chemoradiotherapy; HNC, head and neck cancer; ICD-10, International Classification of Diseases, 10th Revision; PEG, percutaneous endoscopic gastrostomy; RIG, radiologically inserted gastrostomy.

### Baseline characteristics

Following 1:1 propensity-score matching, most recorded demographic, comorbidity, tumor, laboratory, and oncologic covariates met the prespecified balance threshold (SMD < 0.10; Table 1 and Supplementary Table S2). ECOG performance status, however, was available for only 0.5% of patients. In the matched prophylactic–very-late comparison, the observed mean ECOG scores were 1.4 ± 1.1 (*n* = 12) and 1.4 ± 1.2 (*n* = 11), respectively. Because these estimates were based on very small subsets, their apparent similarity was statistically uninformative and should not be interpreted as evidence that performance status was balanced across the full cohorts. ECOG also remained imbalanced in the early- and late-reactive comparisons. Formal tumor stage was documented in only 15.2% of the prophylactic cohort and 14.7% of the very-late cohort. This limited availability prevented meaningful assessment of stage balance, and the propensity-score matching should not be interpreted as adequate adjustment for anatomical stage or tumor burden.

**Table 1:** Baseline characteristics of the propensity-score–matched cohorts—very-late versus prophylactic gastrostomy placement.

Characteristic	Very-late (*n* = 2,251)	Prophylactic (*n* = 2,251)
Age, years (mean ± SD)	66.9 ± 11.3	66.9 ± 10.9
Male	73.8%	73.3%
White	76.3%	76.8%
Black or African American	14.3%	14.0%
Asian	3.4%	3.3%
Hispanic or Latino	5.4%	5.4%
Hypertension	71.7%	72.6%
Ischemic heart disease	39.1%	38.7%
Heart failure	17.5%	16.9%
Atrial fibrillation/flutter	20.2%	19.5%
Cerebrovascular disease	30.7%	30.1%
Malnutrition (baseline)	53.0%	53.8%
Tracheostomy	19.3%	19.3%
Serum albumin, g/dL (mean ± SD)	3.4 ± 0.7	3.3 ± 0.7
ECOG score (mean ± SD)	1.4 ± 1.2	1.4 ± 1.1
HNC site (C00–C14)	79.6%	80.0%
Ill-defined, secondary, or unspecified malignancy (C76–C80)	71.8%	74.4%
Base of tongue	2.0%	2.2%
Other/unspecified tongue	2.3%	2.1%
Other/unspecified mouth	0.8%	0.9%
Tonsil	2.1%	2.1%
Nasopharynx	0.4%	0.4%
Hypopharynx	0.4%	0.4%
Larynx	4.1%	3.8%
Stage 1	2.6%	2.5%
Stage 2	2.7%	2.9%
Stage 3	2.8%	2.9%
Stage 4	6.6%	6.9%

Values are percentages unless stated as mean ± SD. The same matched cohort was analyzed in both the 1–2- and 2–5-year intervals. ECOG performance status was available in only 0.5% of patients (very-late, *n* = 11; prophylactic, *n* = 12). Primary tumor subsite codes were not mutually exclusive. ECOG, Eastern Cooperative Oncology Group; HNC, head and neck cancer; SD, standard deviation.

### Post-gastrostomy overall survival

In the matched prophylactic and very-late cohorts, post-gastrostomy survival did not differ significantly during the 1–2-year interval in the unadjusted analysis (HR 0.90, 95% CI 0.65–1.25, *p* = 0.523) (Figure 2A). The adjusted model estimated a lower post-gastrostomy mortality hazard in the prophylactic cohort [adjusted hazard ratio (aHR) 0.72, 95% CI 0.54–0.96, *p* = 0.027] (Figure 3). During the 2–5-year interval, observed post-gastrostomy survival was higher in the prophylactic cohort (88.4% vs. 83.2%; HR 0.69, 95% CI 0.48–0.98, *p* = 0.038) (Figure 2B), with an aHR of 0.61 (95% CI 0.45–0.83, *p* = 0.001) after adjustment. Among the recorded covariates, older age, palatal primary site during the 1–2-year interval, and hypopharyngeal primary site during the 2–5-year interval were associated with higher post-gastrostomy mortality (Figure 3).

**Figure 2:**
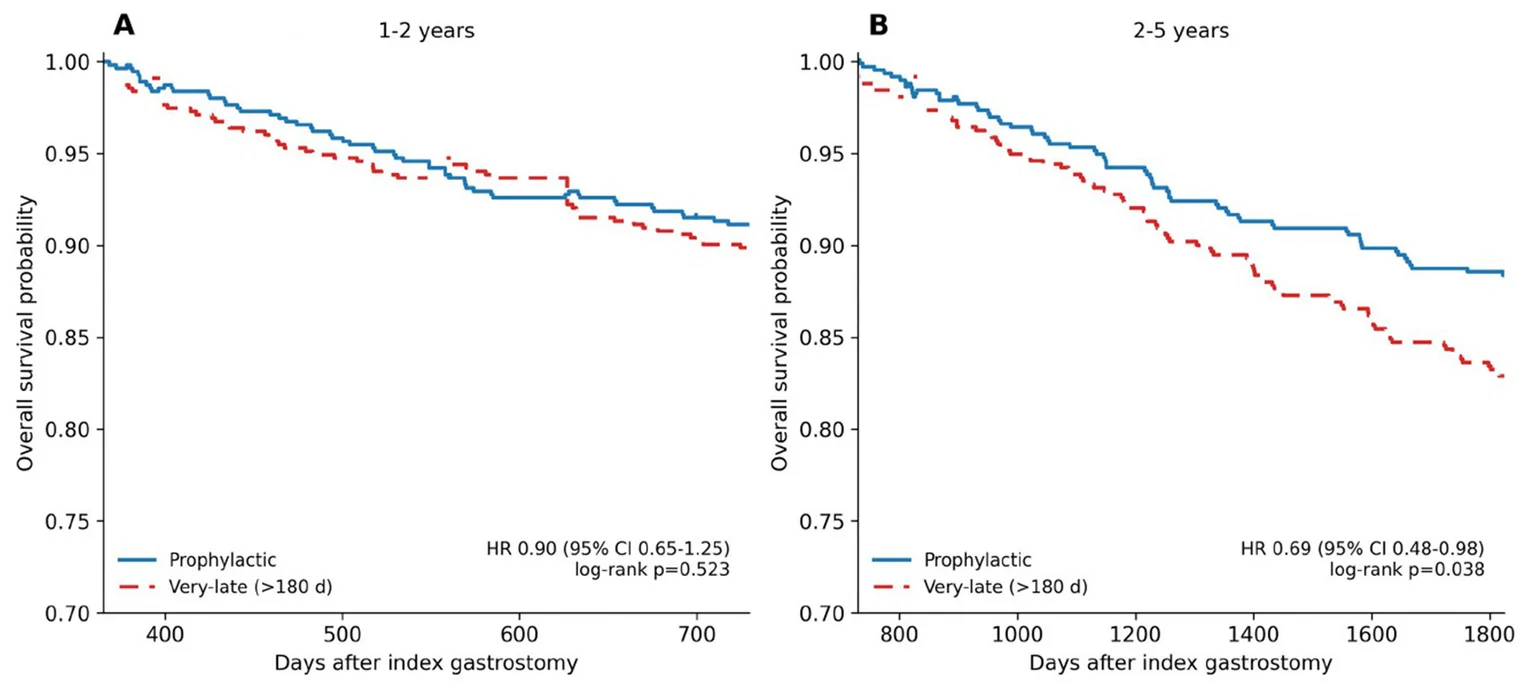
Kaplan–Meier estimates of post-gastrostomy overall survival: Prophylactic versus very-late placement **(A)** 1–2 years; **(B)** 2–5 years. Kaplan–Meier estimates of post-gastrostomy overall survival for the prophylactic (solid blue line) and very-late (dashed red line) cohorts. Time zero was the date of index gastrostomy placement; each panel begins at the start of its follow-up interval. Hazard ratios are expressed for prophylactic relative to very-late placement: 1–2 years, *HR* 0.90 (95% CI 0.65–1.25), log-rank *p* = 0.523; 2–5 years, *HR* 0.69 (95% CI 0.48–0.98), log-rank *p* = 0.038. An *HR* below 1.0 indicates a lower post-gastrostomy mortality hazard in the prophylactic cohort and should not be interpreted as a causal benefit. Curves are based on the matched cohort (*n* = 2,251 per group). The *y*-axis is truncated (0.70–1.00). CI, confidence interval; HR, hazard ratio.

**Figure 3:**
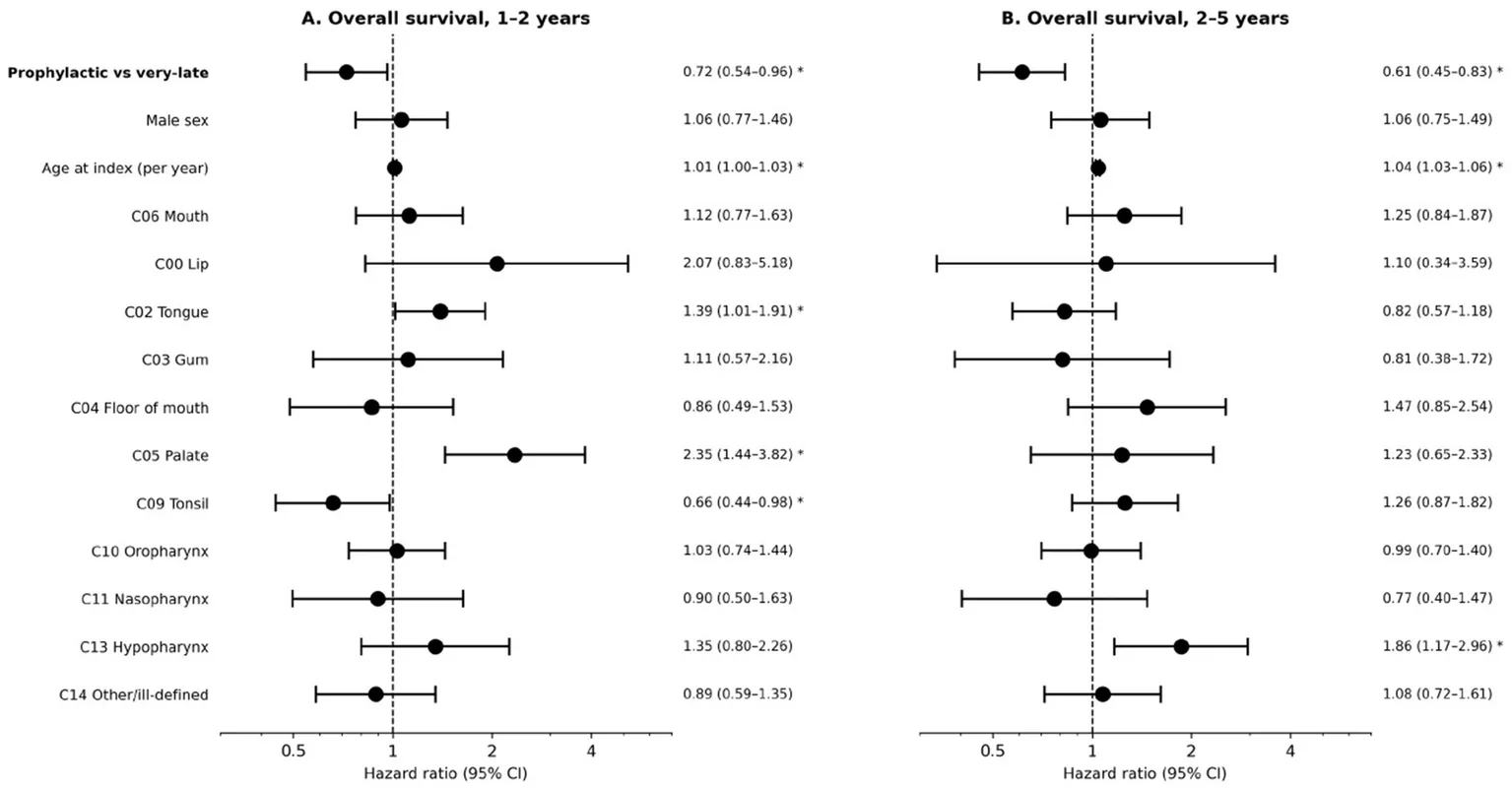
Multivariable Cox proportional-hazards models of post-gastrostomy overall survival: prophylactic versus very-late placement (**A**, 1–2 years; **B**, 2–5 years). Markers are hazard ratios; horizontal bars are 95% confidence intervals, plotted on a logarithmic scale; the dashed line denotes HR = 1.0. An asterisk (*) indicates statistical significance (95% CI excluding 1.0). CI, confidence interval; HR, hazard ratio.

### Exploratory timing-stratified post-gastrostomy survival

Multivariable Cox adjustment for age, sex, and primary tumor site was performed only for the matched prophylactic–very-late comparison, as described above. The other timing comparisons are reported as HRs from the propensity-score-matched cohorts without additional adjustment. Because survival follow-up began at gastrostomy placement, these comparisons describe post-gastrostomy prognosis among patients who survived to placement within their respective timing intervals. They were therefore interpreted as exploratory.

Observed post-gastrostomy survival was similar between the prophylactic and early-reactive cohorts during both intervals (HR 0.94, 95% CI 0.77–1.16, *p* = 0.580 at 1–2 years; HR 0.95, 95% CI 0.77–1.16, *p* = 0.594 at 2–5 years) (Figure 4). During the 1–2-year interval, observed post-gastrostomy survival was higher in the prophylactic cohort than in the late-reactive cohort (84.2% vs. 77.8%; HR 0.70, 95% CI 0.54–0.90, *p* = 0.005), with no significant difference during the 2–5-year interval (HR 0.64, 95% CI 0.32–1.26, *p* = 0.192).

**Figure 4:**
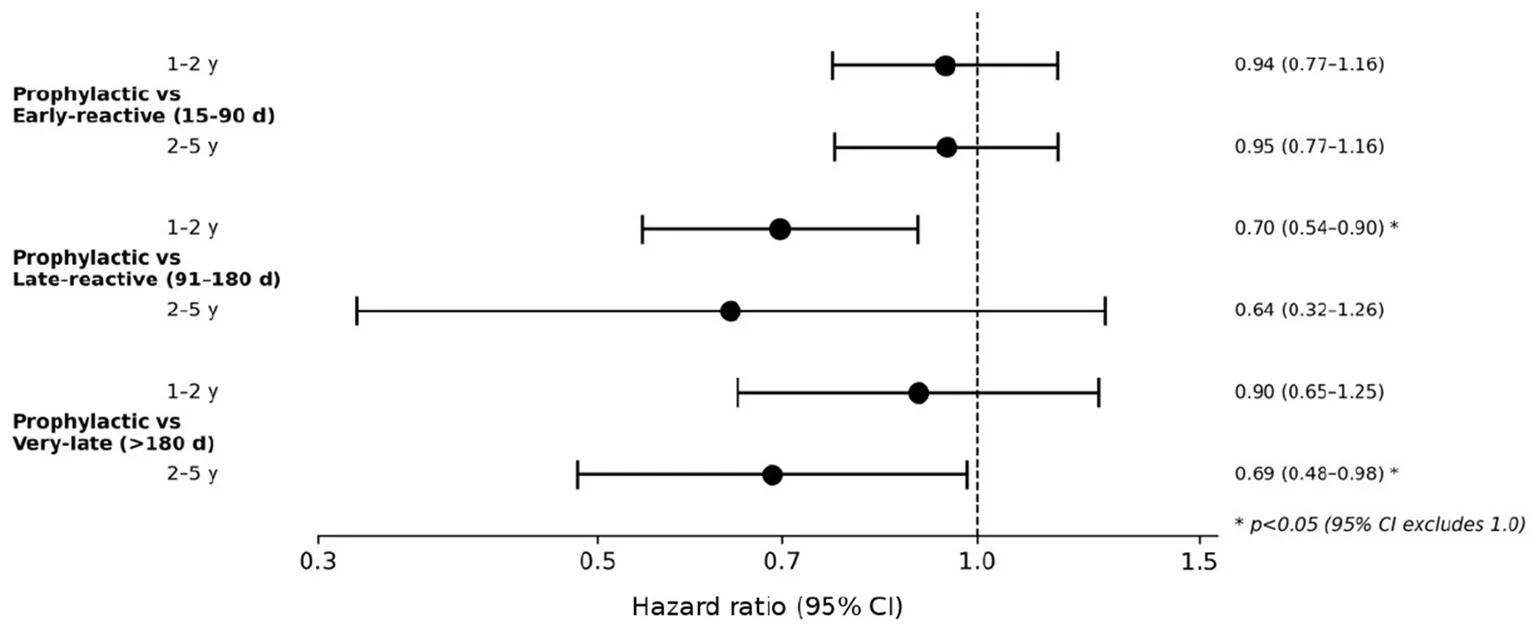
Post-gastrostomy mortality hazard ratios: prophylactic versus later timing groups (exploratory). Each row is a separate 1:1 propensity-score-matched comparison of prophylactic placement (±14 days) with a later timing group, at 1–2 and 2–5 years after gastrostomy. Markers are hazard ratios; horizontal bars are 95% confidence intervals; the dashed line denotes *HR* = 1.0. An asterisk (*) indicates statistical significance (*p* < 0.05; 95% CI excluding 1.0). CI, confidence interval; HR, hazard ratio.

### Secondary outcomes

Compared with the prophylactic cohort, the very-late cohort had higher observed rates of gastrostomy-related complications during both follow-up intervals (3.1% vs. 1.8% and 2.6% vs. 1.3%) and aspiration pneumonia during the 1–2-year interval (1.9% vs. 1.0%). Serum albumin was higher in the prophylactic cohort during the 1–2-year interval (3.89 vs. 3.70 g/dL; all *p* < 0.05). Malnutrition diagnoses (RR 1.39, 95% CI 0.94–2.07), BMI (24.5 vs. 24.8, *p* = 0.45), and emergency department visits (1.86 vs. 1.99, *p* = 0.51) did not differ significantly between the prophylactic and very-late cohorts (Table 2).

**Table 2:** Clinical outcomes of later gastrostomy timing groups compared with prophylactic placement as the reference (propensity-score–matched analyses).

Outcome	Prophylactic vs. early-reactive (15–90 d)		Prophylactic vs. late-reactive (91–180 d)		Prophylactic vs. very-late (>180 d)	
	1–2 y	2–5 y	1–2 y	2–5 y	1–2 y	2–5 y
Malnutrition, RR (95% CI)	1.15 (0.95–1.39)	1.07 (0.88–1.30)	1.69 (1.33–2.13) *	1.00 (0.47–2.15)	1.39 (0.94–2.07)	1.24 (0.83–1.87)
Gastrostomy (PEG) complications, RR (95% CI)	1.06 (0.81–1.40)	1.01 (0.74–1.39)	1.39 (0.94–2.06)	1.27 (0.58–2.79)	1.75 (1.19–2.57) *	2.00 (1.29–3.11) *
Aspiration pneumonia, RR (95% CI)	1.24 (0.93–1.64)	1.01 (0.76–1.34)	1.83 (1.29–2.59) *	–	1.91 (1.14–3.19) *	1.25 (0.73–2.13)
ED visits, mean no.†	2.26 vs. 2.06	2.99 vs. 2.81	2.27 vs. 2.17	3.64 vs. 2.79	1.86 vs. 1.99	2.49 vs. 2.76
Serum albumin, g/dL‡	3.77 vs. 3.80	3.73 vs. 3.76	3.51 vs. 3.64 *	3.94 vs. 4.15 *	3.70 vs. 3.89 *	3.73 vs. 3.87
BMI, kg/m^2^‡	24.7 vs. 24.5	25.3 vs. 25.2	23.7 vs. 24.4	26.3 vs. 26.4	24.5 vs. 24.8	24.4 vs. 25.3

Each comparison is an independent 1:1 propensity-score–matched analysis of one timing group versus prophylactic (±14 days) placement [matched n per group: early-reactive 2,916 (1–2 y)/3,012 (2–5 y); late-reactive 1,137/1,134; very-late 2,251/2,251]. Binary outcomes (malnutrition, gastrostomy complications, aspiration pneumonia) are reported as risk ratios (RR), prophylactic placement as the reference; RR >1.0 indicates a higher rate in the later timing group. * = 95% CI excludes 1.0, or *p* < 0.05 for continuous outcomes. †ED visits are the mean number of visits among patients with ≥1 visit (later timing group vs. prophylactic); no comparison was significant. ‡Serum albumin and BMI are group means (later timing group vs. prophylactic). “–” denotes events below the platform reporting threshold. BMI, body mass index; CI, confidence interval; ED, emergency department; PEG, percutaneous endoscopic gastrostomy; RR, risk ratio. Bold values indicate statistically significant comparisons (95% CI excluding 1.0, or *p* < 0.05 for continuous outcomes).

Compared with the prophylactic cohort, the late-reactive cohort had a higher observed rate of malnutrition during the 1–2-year interval (14.7% vs. 8.7%; RR 1.69, 95% CI 1.33–2.13) and lower serum albumin (3.64 vs. 3.51 g/dL; both *p* < 0.05). These outcomes did not differ significantly between the prophylactic and early-reactive cohorts (Table 2). These comparisons are descriptive and should not be interpreted as causal effects of gastrostomy timing.

## Discussion

In this large, multicenter cohort, observed post-gastrostomy survival was comparable between the prophylactic and early-reactive cohorts. Lower post-gastrostomy mortality was observed in the prophylactic cohort relative to the late-reactive cohort during the 1–2-year interval and the very-late cohort during the 2–5-year interval. However, these comparisons describe prognosis after gastrostomy among patients who survived to placement within different timing intervals; they do not estimate the effect of a feeding-tube strategy assigned at CRT initiation.

A timing-stratified pattern of this kind has not been consistently reported in previous studies (29, 30). Importantly, the prophylactic and very-late cohorts did not differ significantly in recorded malnutrition diagnoses (*p* = 0.10) or BMI (*p* = 0.45), despite prevention of malnutrition being a principal rationale for prophylactic gastrostomy. Although serum albumin was higher in the prophylactic cohort during the 1–2-year interval, albumin is influenced by inflammation and disease severity and is not a specific measure of nutritional status (31). Furthermore, the observed survival difference emerged during the 2–5-year interval, when neither serum albumin nor coded malnutrition differed significantly. The survival association therefore cannot be readily attributed to improved nutritional status and may instead reflect unmeasured differences in cancer recurrence or progression, treatment-related complications, tumor burden, frailty, or the clinical indication for gastrostomy. This inconsistency further supports interpreting the late and very-late survival comparisons as exploratory and noncausal.

Given the observational design, these associations are hypothesis-generating rather than causal. Beyond survival, the prophylactic cohort had lower observed rates of gastrostomy-related complications than the very-late cohort during both intervals and a lower rate of aspiration pneumonia during the 1–2-year interval. However, these differences should not be interpreted as events prevented by prophylactic placement. The groups did not represent interchangeable treatment strategies, and very-late gastrostomy may have been performed as rescue nutritional access in patients with clinical deterioration, disease recurrence or progression, or treatment-related anatomical changes. The lower complication rates in the prophylactic cohort may therefore reflect the elective clinical context of placement and greater patient stability rather than a causal effect of gastrostomy timing.

A major strength of this study is its large size. Prior work has relied on small, single-center cohorts prone to selection bias and limited power. A large federated network is better suited to this question, which is difficult to address at any single institution given the limited number of HNC patients undergoing CRT. It also offers a pragmatic alternative to prospective trials, which face rising costs and slow patient accrual (32). Our heterogeneous, multicenter population allowed propensity-score matching of recorded covariates, improving comparability for characteristics reliably available in TriNetX. However, matching could not address survivor selection, unmeasured confounding, or the extensive missingness in performance status and cancer stage.

This study has several limitations. First, despite PSM, this observational design remains susceptible to residual confounding from unmeasured factors such as frailty, tumor biology, and institution-specific nutritional practices. ECOG performance status was recorded in only 0.5% of patients. Although it was specified as a matching covariate, this extreme missingness severely limited its contribution to propensity-score estimation. The apparent balance among the few patients with recorded ECOG values does not establish comparable performance status across the full cohorts. Second, the survival analyses are vulnerable to immortal-time or guarantee-time bias and survivor-selection bias. To enter the very-late cohort, patients were required to survive for more than 180 days after CRT initiation and subsequently undergo gastrostomy placement, whereas survival follow-up began only on the date of gastrostomy. The timing cohorts therefore had different pre-entry survival requirements, and the reported estimates represent survival conditional on reaching gastrostomy placement within each interval. Propensity-score matching and covariate-adjusted Cox regression cannot correct selection determined by post-baseline survival. Because a valid time-dependent exposure or landmark sensitivity analysis could not be performed, this bias was not quantitatively corrected. The survival findings should therefore not be interpreted as the causal effect of prophylactic versus delayed gastrostomy placement. In addition, the database did not reliably capture the clinical indication, urgency, or intent of gastrostomy placement. We therefore could not distinguish planned delayed placement from rescue gastrostomy performed because of cancer recurrence or progression, treatment-related fibrosis or stenosis, severe dysphagia, functional decline, or palliative-care needs. The late and very-late cohorts may consequently represent clinically distinct patient populations rather than alternative timing strategies, and direct comparisons cannot isolate the independent effect of gastrostomy timing. Third, examining several secondary outcomes increases the chance of false-positive results, so borderline findings (p near 0.05) should be interpreted with caution. Fourth, malnutrition was identified from diagnostic coding rather than validated nutritional screening tools such as the Malnutrition Universal Screening Tool (MUST) or Nutritional Risk Index (NRI), and serum albumin is a non-specific marker, as noted above. These outcomes should therefore be interpreted as healthcare-recorded nutritional correlates rather than direct measures of nutritional status.

TriNetX also lacked more objective measures of nutritional status, including body composition parameters such as skeletal muscle and fat mass, which carry stronger prognostic value than BMI (33). Fifth, formal cancer stage was documented in only approximately 15% of patients, preventing meaningful adjustment for anatomical stage and tumor burden. The C76–C80 code block is heterogeneous and includes ill-defined, secondary, and unspecified malignant sites. Although retained as a recorded matching covariate, these codes cannot substitute for TNM staging or distinguish regional involvement from distant metastatic disease. Therefore, apparent balance in C76–C80 coding does not establish comparable disease extent, and substantial residual confounding by cancer stage and tumor burden may remain. Human papillomavirus (HPV)/p16 status was also unavailable. Because HPV-positive oropharyngeal cancer carries a substantially better prognosis and differs in treatment tolerance, any imbalance in HPV status between timing cohorts could not be assessed or adjusted and may have contributed to the observed survival differences. The dataset also lacked radiotherapy dose, surgery, prior nasogastric tube use, and patient-reported outcomes such as quality of life and swallowing function.

## Conclusion

Among patients with head and neck cancer undergoing CRT who received gastrostomy, prophylactic and early-reactive placement were associated with comparable post-gastrostomy survival. Patients receiving late or very-late gastrostomy had poorer post-gastrostomy outcomes, and the very-late cohort experienced more gastrostomy-related complications, suggesting that late and very-late placement may identify a clinically higher-risk population. However, because the later timing groups were defined by post-baseline survival and may reflect disease recurrence, progression, treatment-related complications, or functional decline, these findings do not establish that prophylactic gastrostomy improves survival. The results support close nutritional monitoring and individualized, timely enteral access, while prospective studies using a target trial emulation framework are needed to clarify the effect of gastrostomy timing.

## Data Availability

The original contributions presented in the study are included in the article/Supplementary material, further inquiries can be directed to the corresponding author.
