# Supplementary material for: Timing matters: gastrostomy placement and outcomes in patients with head and neck cancer undergoing chemoradiotherapy
Source: Front Nutr. 2026 Sep 18;13:1885057. doi: 10.3389/fnut.2026.1885057 (PMC13630608; doi:10.3389/fnut.2026.1885057)
Supplement: Supplementary file 1 [file Table_1.docx]

**Supplementary File 1: RECORD Checklist**

This study is reported in accordance with the REporting of studies Conducted using Observational Routinely-collected health Data (RECORD) statement, an extension of the STROBE statement. Page numbers refer to the main manuscript; Tables S1–S2 are provided in the Supplementary Material, and Tables 1–2 and Figures 1–4 in the main tables/figures file.

| **Item** | **RECORD checklist item** | **Location in manuscript** |
| --- | --- | --- |
| **Title and Abstract** | | |
| **1** | (a) Indicate the study design with a commonly used term in the title or abstract. (b) Provide an informative and balanced summary in the abstract. | Abstract (p. 2) |
| **RECORD 1.1** | The type of data used should be specified; when possible, the name of the database(s) should be included in the title or abstract. | Abstract (p. 2; TriNetX Collaborative Network) |
| **RECORD 1.2** | If applicable, the geographic region and timeframe should be reported. | Methods — Study Population (p. 6; US network, 2010–2026) |
| **RECORD 1.3** | If linkage between databases was conducted, this should be stated. | Not applicable — single federated network; no linkage |
| **Introduction** | | |
| **2** | Background/rationale. | Introduction (p. 4) |
| **3** | Objectives, including any prespecified hypotheses. | Abstract (p. 2); Introduction (p. 4) |
| **Methods** | | |
| **4** | Study design. | Materials & Methods (p. 5) |
| **5** | Setting, locations, and relevant dates. | Materials & Methods (p. 5); Study Population (p. 6) |
| **6** | Participants: eligibility criteria, sources, and selection. | Study Population (p. 6) |
| **RECORD 6.1** | Detailed methods of population selection (codes/algorithms). | Study Population (p. 6); Table S1 |
| **RECORD 6.2** | Validation of codes/algorithms, if performed. | Methods (p. 5); standard ICD-10/CPT/TNX terminologies (refs 21–24); no additional internal validation |
| **RECORD 6.3** | Flow diagram or descriptive table of population selection. | Figure 1; Results (p. 8) |
| **7** | Variables: outcomes, exposures, confounders, effect modifiers. | Covariates and Matching (pp. 6–7); Outcomes (p. 7) |
| **RECORD 7.1** | Complete list of codes/algorithms used to classify variables. | Table S1 |
| **8** | Data sources/measurement. | Methods (p. 5); Outcomes (p. 7); Table S1 |
| **9** | Efforts to address sources of bias. | Covariates and Matching — PSM (p. 7); Discussion — Limitations (p. 11) |
| **10** | Study size. | Results (p. 8); Statistical Analysis (p. 7) |
| **11** | Quantitative variables: how handled in analyses. | Study Population (p. 6); Statistical Analysis (p. 7) |
| **12** | Statistical methods. | Statistical Analysis (p. 7) |
| **RECORD 12.1** | Extent of investigator access to the database population. | Methods (p. 5); Data Sharing (p. 13) |
| **RECORD 12.2** | Data-cleaning methods. | Statistical Analysis (p. 7) |
| **RECORD 12.3 / 13.1** | Linkage across databases and methods/quality of linkage. | Not applicable — single federated network; no linkage |
| **Results** | | |
| **13** | Participants: numbers at each stage; consider a flow diagram. | Results (p. 8); Figure 1 |
| **14** | Descriptive data. | Table 1; Baseline Characteristics (p. 8) |
| **RECORD 14.1** | Extent to which numbers with missing data differed from complete cases. | Baseline Characteristics (p. 8; ECOG 0.5%); denominators in Table 2 |
| **15** | Outcome data. | Table 2; Results (pp. 8–9) |
| **16** | Main results: adjusted estimates and confidence intervals. | Overall Survival (p. 9); Figures 2–3; Table 2 |
| **17** | Other analyses (subgroups, sensitivity). | Timing-Stratified Survival (p. 9); Figure 4. No sensitivity analysis was performed (see Limitations). |
| **Discussion** | | |
| **18** | Key results. | Discussion (p. 9) |
| **19** | Limitations. | Discussion — Limitations (p. 11) |
| **RECORD 19.1** | Implications of using data not created to answer the research question (misclassification, unmeasured confounding, missing data, changing eligibility). | Discussion — Limitations (p. 11) |
| **20** | Interpretation. | Discussion (pp. 9–12); Conclusions (p. 12) |
| **21** | Generalisability. | Discussion — strengths (p. 10) |
| **Other Information** | | |
| **22** | Funding and role of funders. | Funding (p. 13) |
| **RECORD 22.1** | How to access supplemental information (protocol, raw data, or code). | Data Sharing (p. 13); Table S1 |

*Reference: Benchimol EI, Smeeth L, Guttmann A, et al. The REporting of studies Conducted using Observational Routinely-collected health Data (RECORD) statement. PLoS Med. 2015;12(10):e1001885.*

**Supplementary Table S1.** Diagnostic, procedural, and laboratory codes used for cohort definition, matching covariates, and outcomes.

| **Variable** | **Code(s)** |
| --- | --- |
| **Cohort definition** | |
| Head and neck cancer | C00–C14, C32 |
| Chemotherapy | TNX:1002, TNX:100211 |
| Radiation therapy | TNX:1001; ICD-10-PCS D; CPT 77412, 77427, 1010875, 1022242, 1035176, 1035663 |
| Gastrostomy (PEG/RIG) | CPT 49440; ICD-10-PCS 0DH6, 0DH63UZ, 0DH64UZ |
| **Matching covariates** | |
| Age, sex, self-reported race and ethnicity | — |
| Hypertensive disease | I10–I15 |
| Ischemic heart disease | I20–I25 |
| Heart failure | I50 |
| Atrial fibrillation/flutter | I48 |
| Cerebrovascular disease | I60–I69 |
| Malignant neoplasms, lip/oral cavity/pharynx | C00–C14 |
| Malignant neoplasms, ill-defined/secondary/unspecified | C76–C80 |
| Tracheostomy status | Z93.0 |
| Malnutrition | E40–E46 |
| Primary tumor subsites | C01, C02, C06, C09, C11, C13, C32 |
| Cancer stage | Stage III, Stage IV |
| Serum albumin | TNX:9045 |
| C-reactive protein | TNX:9063 |
| ECOG performance status | TNX:89247-1 |
| **Outcomes** | |
| Overall survival (deceased) | Demographics: Deceased |
| Malnutrition | E40–E46 |
| Gastrostomy-related complications | K94.2 |
| Aspiration pneumonia | J69.0 |
| Emergency department visits | CPT 99283, 99284, 1013711 |
| Serum albumin | TNX:9045 |
| Body mass index | TNX:9083 |

*All diagnostic codes are ICD-10-CM; procedure codes are CPT or ICD-10-PCS; laboratory, BMI, chemotherapy, and radiation terms use TriNetX (TNX) identifiers. Prophylactic, reactive, and delayed placement were distinguished by the timing of gastrostomy relative to CRT initiation (see Methods), using the same procedure codes.*

*Abbreviations: CPT, Current Procedural Terminology; ECOG, Eastern Cooperative Oncology Group; ICD-10-CM, International Classification of Diseases, Tenth Revision, Clinical Modification; ICD-10-PCS, ICD-10 Procedure Coding System; PEG, percutaneous endoscopic gastrostomy; RIG, radiologically inserted gastrostomy.*

**Supplementary Table S2.** Distribution of documented head and neck cancer subsites in the propensity-score–matched cohort (prophylactic vs. very-late placement).

| **Tumor subsite (ICD-10)** | **Very-late (n=2,251)** | **Prophylactic (n=2,251)** |
| --- | --- | --- |
| Lip, oral cavity, and pharynx (C00–C14) | 1,791 (79.6) | 1,800 (80.0) |
| Base of tongue (C01) | 44 (2.0) | 50 (2.2) |
| Other and unspecified parts of tongue (C02) | 52 (2.3) | 47 (2.1) |
| Other and unspecified parts of mouth (C06) | 19 (0.8) | 20 (0.9) |
| Tonsil (C09) | 47 (2.1) | 48 (2.1) |
| Nasopharynx (C11) | 10 (0.4) | 10 (0.4) |
| Hypopharynx (C13) | 10 (0.4) | 10 (0.4) |
| Larynx (C32) | 93 (4.1) | 85 (3.8) |

*Values are number (%), after 1:1 propensity-score matching. All covariates were balanced after matching (all standardized mean differences <0.10). Subsite codes within the C00–C14 group are not mutually exclusive and do not sum to the C00–C14 total, as many patients carried only the broader category code; subsite classification was limited by EHR coding granularity, and subsite-stratified outcome analyses were underpowered after matching (see Limitations). Counts below 10 are suppressed per the TriNetX minimum reporting threshold. Abbreviations: EHR, electronic health record; ICD-10, International Classification of Diseases, 10th Revision.*
